# Cellular and molecular features related to exceptional therapy response and extreme long‐term survival in glioblastoma

**DOI:** 10.1002/cam4.5681

**Published:** 2023-02-12

**Authors:** B. Decraene, M. Vanmechelen, P. Clement, J. F. Daisne, I. Vanden Bempt, R. Sciot, A. D. Garg, P. Agostinis, F. De Smet, S. De Vleeschouwer

**Affiliations:** ^1^ KU Leuven, Laboratory for Precision Cancer Medicine Translational Cell and Tissue Research Unit Leuven Belgium; ^2^ KU Leuven Department of Neurosciences Experimental Neurosurgery and Neuroanatomy Research Group Leuven Belgium; ^3^ Department of Neurosurgery University Hospitals Leuven Leuven Belgium; ^4^ Department of General Medical Oncology University Hospitals Leuven Leuven Belgium; ^5^ Radiation Oncology Department University Hospitals Leuven Leuven Belgium; ^6^ Department of Human Genetics University Hospitals Leuven Leuven Belgium; ^7^ Department of Pathology University Hospitals Leuven Leuven Belgium; ^8^ KU Leuven, Laboratory of Cell Stress & Immunity (CSI), Department of Cellular & Molecular Medicine Leuven Belgium; ^9^ Cell Death Research and Therapy Group Department of Cellular and Molecular Medicine, KU Leuven, Herestraat 49, 3000 Leuven, Belgium VIB Center for Cancer Biology, 3000 Leuven Belgium; ^10^ KU Leuven Leuven Brain Institute (LBI) Leuven Belgium

**Keywords:** gbm, glioblastoma, long‐term survivors, molecular markers

## Abstract

Glioblastoma Multiforme (GBM) remains the most common malignant primary brain tumor with a dismal prognosis that rarely exceeds beyond 2 years despite extensive therapy, which consists of maximal safe surgical resection, radiotherapy, and/or chemotherapy. Recently, it has become clear that GBM is not one homogeneous entity and that both intra‐and intertumoral heterogeneity contributes significantly to differences in tumoral behavior which may consequently be responsible for differences in survival. Strikingly and in spite of its dismal prognosis, small fractions of GBM patients seem to display extremely long survival, defined as surviving over 10 years after diagnosis, compared to the large majority of patients. Although the underlying mechanisms for this peculiarity remain largely unknown, emerging data suggest that still poorly characterized both cellular and molecular factors of the tumor microenvironment and their interplay probably play an important role. We hereby give an extensive overview of what is yet known about these cellular and molecular features shaping extreme long survival in GBM.

## Graphical abstract

A comprehensive overview of cellular and molecular features related to exceptional therapy response and extreme long‐term survival in glioblastoma patients.

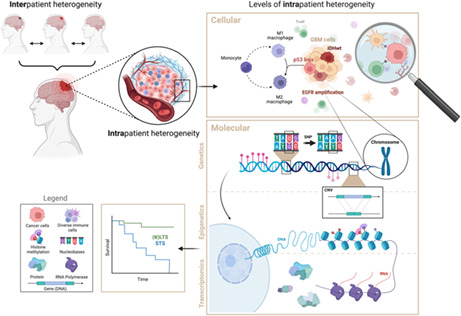

## 1 INTRODUCTION

Glioblastoma Multiforme (GBM) remains the most common malignant primary brain tumor with a dismal prognosis that rarely exceeds beyond 2 years of survival post‐diagnosis, despite extensive therapy, which consists of maximal safe surgical resection, radiotherapy, and/or chemotherapy.^1^ Recently, it has become clear that GBM is not one homogeneous entity and that both intra‐ and intertumoral heterogeneity contribute significantly to differences in tumoral behavior, which may consequently be responsible for differences in survival.^2^, ^3^, ^4^ Moreover, tumor heterogeneity is one of the barriers to long‐term therapeutic efficacy and as such represents an area of intense investigation. Strikingly and despite its dismal prognosis, small fractions of GBM patients seem to display extended survival compared to most patients. However, the underlying mechanisms for this peculiarity remain largely unknown, even though emerging data suggests that both cancer cell‐autonomous and microenvironmental factors and their interplay likely play an important role. As stated further, the aim of this work is to present a concise summary of the current state‐of‐the‐art on this striking topic.

Long‐term survival is often defined as a survival time of more than 2 or 3 years from diagnosis.^5^, ^6^ However, from a patient's perspective, we would rather define long‐term survivorship (LTS) in GBM as patients who survive at least 5 years (60 months) after diagnosis, as opposed to short‐term survivors (STS). The latter can be described in many ways. We define STS as patients who survive <36 months unless mentioned otherwise. Anecdotical cases have been published of patients surviving longer than 10 years, which may ultimately turn out to be a different subtype of GBM or another CNS malignancy that behaves uniquely.^7^, ^8^ To make the distinction with 5‐year survivors, we will further address these cases as extreme long‐term survivors (eLTS), estimated to be <1% of all GBM patients.^9^ Finally, we would also like to define a separate subgroup of patients, namely exceptional responders. In literature, exceptional responders are often defined as patients achieving a unique, partial, or complete, response after non‐surgical therapies (mostly in the context of experimental treatment modalities) that are barely effective for most other patients, which is only seen in about 10% of GBM patients when it is sustained for a longer period of time.^10^ This definition of exceptional response implies the use of highly specific and targeted drugs and would thus require an extensive description of all possible (experimental) treatment methods used in these patients, their working mechanisms, and interactions, which is beyond the scope of this review. Nevertheless, we conducted a literature search to discover cellular and molecular factors that could independently predispose a GBM to exceptional therapy response, in addition to and beyond the exact therapy given.

Some authors suggest that such exceptional observations (exceptional therapy response and survival) may also be related to misclassifications of low‐grade glioma or may be attributed to statistical errors.^5^, ^11^, ^12^

Several clinical variables have been associated with patient survival in GBM, with age, Karnofsky performance score (KPS), and extent of resection being the most consistent.^6^, ^13^ With advances in multi‐omics technologies and disease diagnostics (such as genomics, bulk, and single‐cell transcriptomics, DNA methylation profiling, and next‐generation sequencing), and their integration with disease diagnostics, numerous prognostic molecular markers have been proposed. So far, only O6‐methylguanine‐DNA‐methyltransferase (*MGMT*) gene promotor hypermethylation and isocitrate dehydrogenase (*IDH*) gene mutations, could be identified as robust, well‐established biomarkers that are linked to outcome and therapy response. However, integrated multi‐level analysis aimed at identifying novel biomarkers or combinations of biomarkers associated with exceptional therapy response and/or (extreme) long‐term survival is still largely missing.

To this end, the main goal of this biomedical literature review is to shed a broader light on the still unknown cellular and molecular features, as well as clinicopathological correlates and possible mechanisms behind (extreme) long‐term survival of GBM patients. This is in order to pave the way for a consolidated conclusion on this important topic.

## 2 METHODS

### 2.1 Study design

A literature search was conducted, focusing on research articles and review papers containing cellular and molecular information from (extreme) long‐term surviving patients or exceptional responders (implying long progression‐free survival), as defined in the introduction. Since both groups are not perfectly interchangeable, we make a clear distinction between the two, focusing primarily on exceptional survival, as explained earlier. Studies focusing exclusively on case reports of the exceptional response of experimental therapies or non‐human studies were excluded, as well as pediatric cases since these tumors should be considered a separate pathology.

### 2.2 Literature search

**FIGURE 1:**
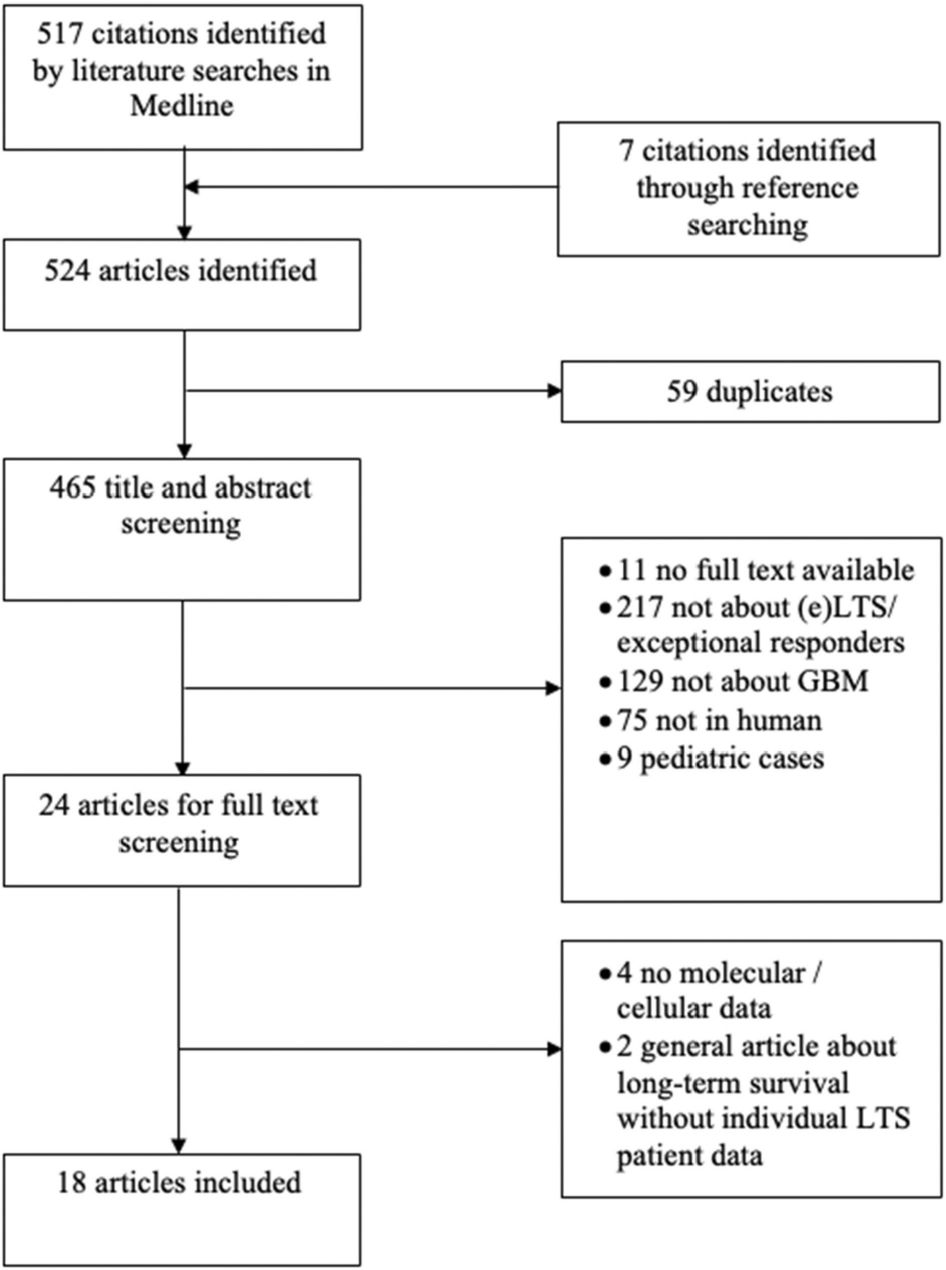
Search strategy.

An online literature search in Medline was performed for relevant articles from inception to November 2021, using a combination of the following search strategies: (1) ‘exceptional responders’ AND ‘glioblastoma’ (2) ‘long‐term response’ AND ‘glioblastoma’ (3) ‘molecular’ AND ‘long‐term’ AND ‘glioblastoma’ (4) ‘macrophage’ AND ‘long‐term’ AND ‘glioblastoma’ (5) ‘t‐cell’ AND ‘long‐term’ AND ‘glioblastoma’ (6) ‘exceptional’ AND ‘therapy’ AND ‘response’ AND ‘glioblastoma’ (Figure 1). For each individual search, both MeSH terms and free‐words searches were used. We further expanded our search by using the ‘related article’ function and by including references from the initial selection. We removed duplicates and screened both title and abstract. Potentially relevant articles were included for full‐text reading. Studies in English, French, German, and Dutch were screened for inclusion. When data from the same study population were published several times, the most recent version was withheld. In studies in which only a subpopulation of their patient population was an (e)LTS, we looked specifically at the outcomes for those patients. If it was not possible to look at them separately, and thus the results were not specific for (e)LTS, the study was excluded. Studies for which only an abstract, and thus insufficient data, were available were also excluded. Finally, since we focused on cellular and molecular data related to these (e)LTS, a lack of this information naturally also led to exclusion.

### 2.3 Outcome

**FIGURE 2:**
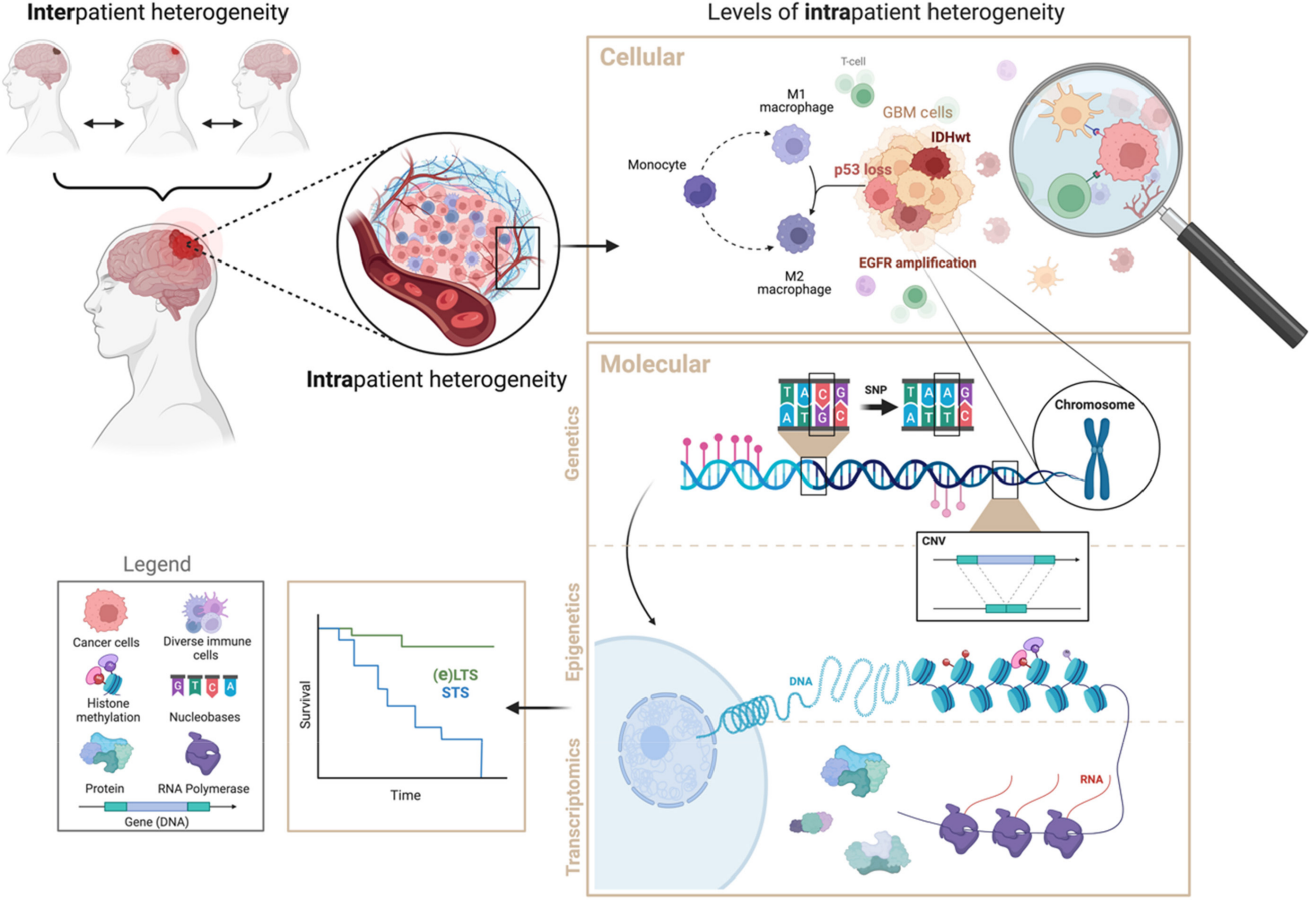
Next to interpatient heterogeneity, intrapatient heterogeneity is observed on multiple levels in GBM. Variations in cellular features (tumor microenvironment composition and interactions) and molecular features (single nucleotide polymorphisms, copy number variation and alterations, somatic mutations, and epigenetic changes) contribute to this intrapatient heterogeneity and subsequently to differences in tumor behavior leading to distinct survival curves in GBM patients.

We investigated the potentially distinct cellular and molecular profiles of eLTS and exceptional responders compared to STS. We will further divide the molecular changes into the different steps of protein formation: genetics, transcriptomics, and epigenetics. The protein changes themselves are discussed throughout the sections as well as in the cellular section. Figure 2 shows the concept we want to elucidate in this article; how intra‐ and interpatient heterogeneity at both cellular and molecular levels could contribute to differences in survival in GBM patients. Therefore, data at the cellular level and from different genomic layers were collected: mutation analysis, structural and copy number variations (CNV) analysis, as well as epigenetic information. A descriptive overview of the results is given in the following paragraphs.

We focused on IDH wild type (IDHwt) GBM, as IDH mutant (IDHmt) tumors are known to have a better prognosis and should no longer be considered GBM according to the latest WHO 2021 CNS tumor classification.^14^ We will therefore report when features were only significant or investigated in IDHmt GBM. Although several clinical and radiological parameters such as the extent of resection, age, and gender may influence survival and therapy response, we will not focus on these characteristics because our focus is on investigating cellular and/or molecular components. We assume that studies that focus on nonclinical parameters are standardized as much as possible for these parameters among their included cases. If not, or if there is any doubt, we will mention this in our review. Secondly, we only looked for patients who survived for at least 5 years. By limiting our results to this population, it is more likely that we are dealing with causally distinct underlying cellular or molecular mechanisms rather than purely clinical parameters as an explanation for exceptional response or survival.

## 3 RESULTS

### 3.1 Search results

Eighteen articles were identified describing the cellular or molecular features of (e)LTS (Table 1); 14 articles described newly diagnosed, primary GBM. Of the remaining 4 articles, two described a mixture of both primary and secondary GBM (but clearly distinguished between the two groups in their description),^15^, ^16^ and two did not mention whether the GBM included were primary or secondary, nor whether the analysis was performed on newly diagnosed or recurrent samples.^17^, ^18^

**TABLE 1:** Studies included in this review.

Article	Study design	Number of (e)LTS	Number of exceptional responders	Analyses	Cellular/ molecular characteristic	Significance/remarks
Donson et al.^18^ Increased Immune Gene Expression and Immune Cell Infiltration in High Grade Astrocytoma Distinguish Long from Short‐Term Survivors Aug. 2012	Comparative	6 LTS	/	Gene expression Ontology analysis	Both T‐cell and myeloid lineage‐associated genes + immune cell infiltration +	Increased immune cell infiltration is linked to LTS
Anghileri et al.^78^ High tumor mutational burden and T‐cell activation are associated with long‐term response to anti‐PD1 therapy in Lynch syndrome recurrent glioblastoma patient Nov. 2020	Case report	1 LTS	1 (same patient)	Whole exome sequencing 2 IHC 3 Flow cytometry on the peripheral blood	/	Hypermutator phenotype associated with germinal mutations of MMR genes and abundant T‐cell infiltration, leading to a better response to Anti‐PD1 therapy
Ferguson et al.^120^ A validated integrated clinical and molecular glioblastoma long‐term survival‐predictive nomogram Oct. 2020	Cohort	21 LTS	/	Gene expression	*PTEN* mutations ‐	/
Sonoda et al.^17^ Long‐term survivors of glioblastoma: clinical features and molecular analysis May 2009	Comparative	7 LTS	/	Gene expression 2 Immuno‐histochemistry (IHC)	*MGMTp methylation* in 4/7 LTS patients, and 5/16 STS patients (not significant with *p*‐value 0,18)	No difference in Ki67 expression, *PTEN* mutation or *TP53* mutation between STS and LTS
Jiang et al.^16^ Differential Predictors and Clinical Implications Associated With Long‐Term Survivors in IDH Wildtype and Mutant Glioblastoma May 2021	Comparative	44 LTS (27 IDHmt)	/	FISH 2 Sequencing data 3 IHC	Higher rate of *MGMTp* methylation in LTS	/
Lu et al.^107^ Molecular Predictors of Long‐Term Survival in Glioblastoma Multiforme Patients Apr. 2016	Cohort	29 LTS*	/	Mutation sequencing Gene expression methylation profiling Copy number analysis miRNA expression	MiR‐222 + Oncogene *DUSP28* – *NEUROG1* +	Absence of unique LTS profiles and the lack of similarity between LTS GBM and LGG
Marton et al.^109^ Over ten years overall survival in glioblastoma: A different disease? Jan. 2020	Comparative	7 eLTS (of which 4 IDHmt)	/	Gene expression HC	*MGMTp* methylation in 85.7% No significant difference in *TERTp* and *ATRX* mutation	*MGMTp* methylation is associated with LTS
Martinez et al.^114^ Independent Molecular Development of Metachronous Glioblastomas with Extended Intervening Recurrence‐free Interval Apr. 2006	Case report	1 LTS	/	IHC Gene expression	*TP53* and *PTEN* mutations EGFR normal	/
Das et al.^115^ A clinicopathological and molecular analysis of glioblastoma multiforme with long‐term survival Jan. 2011	Cohort	1 LTS	/	Methylation profiling 3 IHC	*MGMTp* methylation was present. Strong p53 and weak PTEN immunostaining were present.	High *MGMTp* methylation and PTEN expression linked to LTS
Kuhajda et al.^116^ Twenty‐year survival in glioblastoma: a case report and molecular profile Nov. 2009	Case report	1 eLTS	/	Gene expression	*MGMTp* methylated, PTEN and p53 positive	Triple positivity is uncommon, but may be related to LTS
Sabel et al.^117^ Long‐term survival of a patient with giant cell glioblastoma Apr. 2001	Case report	1 eLTS	/	IHC Gene expression	No EGFR amplification Point mutation in *TP53*	IDH status not mentioned
Deb et al.^121^ Glioblastoma multiforme with long term survival Mar. 2005	Cohort	5 LTS	/	Gene expression	p53 positive in 4/5 cases p27 negative in 5/5 EGFR negative in 5/5 P16 negative in 3/5	/
Burgenske et al.^127^ Molecular profiling of long‐term IDH‐wildtype glioblastoma survivors Nov. 2019	Comparative	12 LTS	/	Copy number analysis Methylation profiling Gene expression Mutation sequencing	Sphingomyelin metabolism + *MGMTp* methylation + (Classic GBM mutations and copy number changes)	Decreased GBM growth, invasion, and angiogenesis in LTS
Fazi et al.^138^ The transcriptome and miRNome profiling of glioblastoma tissues and peritumoral regions highlights molecular pathways shared by tumors and surrounding areas and reveals differences between short‐term and long‐term survivors Sep. 2015	Comparative	1 LTS	/	Gene expression 2 MicroRNA deep sequencing	Only outcome data for the whole patient group (living >36 months) is available.	/
Cantero et al.^15^ Molecular Study of Long‐Term Survivors of Glioblastoma by Gene‐Targeted Next‐Generation Sequencing Aug. 2018	Comparative	7 LTS	/	Gene expression	PI3K pathway alterations +	*PDGFRA* alteration is suggested to be a favorable molecular factor
Nakagawa et al.^131^ Clinical and molecular prognostic factors for long‐term survival of the patients with glioblastomas in a single institutional consecutive cohort Jun. 2017	Comparative	7 LTS	/	Gene expression	*MGMTp* methylation no effect in LTS	*MGMTp* methylation is linked to longer survival up to three years, but not exceptional survival
Morita et al.^156^ Long‐term survivors of glioblastoma multiforme: clinical and molecular characteristics Mar. 1996	Cohort	10 LTS	/	Gene expression	EGFR +	Overexpression of EGFR does not preclude long‐term survival
Hartmann et al.^157^ Long‐term survival in primary glioblastoma with versus without isocitrate dehydrogenase mutations Aug. 2013	Comparative	20 LTS IDH wt (13 IDH mt not included)	/	Methylation profiling Gene expression	LTS had less frequent *TP53* mutations compared with 3‐year survivors (17,5%) 59% *MGMTp* methylation, 33% in STS IDHwt 35% EGFR amplification, 46% in STS IDHwt 10,5% *TP53* mutation, 14,5% in STS IDHwt	/

*Note*: ‘+’ = increased; ‘−’= decreased.

* In this study a 4.5‐year cut‐off instead of a 5 year cut‐off was used.

It has been hypothesized that the survival of therapy‐resistant cellular subpopulations within a newly diagnosed GBM could be responsible for recurrence.^19^ Subsequently, these subpopulations tend to dominate the composition of the recurrent tumor over time.^20^ It is reasonable to assume that cellular or molecular features important for survival, therefore, tend to become more pronounced upon recurrence. However, these compositional changes in the tumor micro‐environment are also highly dependent on the therapy given.^21^ In our opinion, the distinction between primary and secondary GBM is more important for prognosis. Since secondary GBM, arising from a low‐grade glioma, is more often associated with IDH mutations, they are generally associated with better survival.^22^, ^23^ This article will describe prognostic markers for primary GBM unless otherwise noted.

Since articles describing cellular and molecular factors in (e)LTS patients are scarce and mostly limited to case reports, we will further complement the findings of the 18 (e)LTS ‐containing articles with additional prognostic factors for longer survival documented in the literature. We will clearly describe whether a feature applies specifically to (e)LTS or is linked to prolonged survival.

Furthermore, only one article described cellular or molecular features of exceptional responders with individual patient data. This subgroup is described in a separate subsection.^18^

### 3.2 Cellular features of the tumor microenvironment associated with long‐term survival

#### 3.2.1 The main cellular players—introduction

GBM is a low‐antigenic and non‐immunogenic tumor with severe depletion of lymphocytes.^24^ Tumor‐associated macrophages (TAM) constitute the largest non‐neoplastic cell population in the tumor microenvironment (TME) of GBM. In GBM, they consist mainly of bone‐marrow‐derived myeloid cells (monocytes), in addition to the brain‐resident microglia. Although TAM are considered molecularly heterogeneous, they generally promote tumor progression; apart from the ability to suppress T‐cell function, they also interact with the innate immune system. Here, they sense hypoxic conditions and secrete pro‐angiogenic and pro‐mitogenic factors in response.^25^, ^26^, ^27^ Two different phenotypes, with a whole continuum in between, were identified: the M1 (pro‐inflammatory) and M2 (anti‐inflammatory) subtypes. The latter is said to be more prominent in GBM.^28^ It was also reported that macrophages induce transition toward a Mesenchymal‐like cancer state.^29^ Moreover, TAM (mainly M2) contributes to chemo‐and radiotherapy resistance by induction of regenerative programs, of which the exact underlying mechanisms are not yet known.^30^

The second largest non‐cancerous cells in the GBM TME are T‐cells, accounting for <10% of all cells. Tumor cells tend to induce quantitative and qualitative T‐cell dysfunction leading to T‐cell aging, tolerance, and exhaustion.^31^, ^32^ Other factors also play a role; e.g., the inability of antigen‐presenting cells (APC) to present tumor antigen and subsequentially activate T‐cells; or the infiltration of immunosuppressive regulatory T‐cells (Treg) and Myeloid‐derived suppressor cells (MDSC).^33^ Treg were found to expand in response to GBM‐related factors.^34^ Furthermore, hypoxia‐inducible factor 1 alfa (HIF1α) which is expressed by glioma cells in response to oxygen‐depleted conditions, induces Treg migration into the TME of GBMs. Therefore, this phenomenon is best observed at a distance from blood vessels, as blood vessels are responsible for maintaining adequate tissue oxygenation.^35^

MDSC represent around 5% of all cells in human GBM.^36^ Their main function is to suppress immune cells, mainly T‐cells and to a lesser extent B‐cells, dendritic cells (DC) cells, macrophages (skewing these cells toward a M2 subtype) and Natural killer (NK) cells.^37^, ^38^, ^39^, ^40^ This occurs, for example, through the upregulation of indoleamine 2,3‐dioxygenase (IDO) in MDSC and tumor cells, leading to tryptophan depletion and impairing cytotoxic T‐cell function and survival.^41^, ^42^ Stimulation of Treg and regulatory B‐cells by MDSC has also been described in GBM.^43^, ^44^ Lastly, they promote tumor growth by influencing angiogenesis, invasion, and cancer cell stemness.^45^ In glioma, MDSC density increases during tumor progression and is thus correlated with survival.^46^ The mononuclear MDSC (M‐MDSC), the subpopulation predominantly present in GBM, directly suppresses T‐cell function or induces Treg formation via various factors such as IL‐10 and TGF‐beta.^47^ MDSC infiltration into the TME is therefore associated with worse outcomes in GBM patients.^48^

The exact function of DC, another minor immune cell population, in GBM is still under investigation.^49^ They can be divided into three groups: Langerhans cells, interstitial DCs, and plasmacytoid DCs (the latter one expressing CD123 and CD303).^50^ The first two are grouped because they have myeloid precursors. Therefore, they are called myeloid DCs, expressing CD141 and CD1c.^51^ A complex interplay between DC, other immune cells, and tumor cells has been suggested, with a prominent role in the presentation of tumor antigens leading to the recruitment and stimulation of T‐cells (and NK cells).^52^, ^53^, ^54^, ^55^ Several factors can make DCs a regulatory subtype, which in its turn downregulates CD8+ T‐cell influx and upregulates Treg activation.^56^, ^57^, ^58^

#### 3.2.2 The main cellular players— myeloid‐derived cells

Tumor‐associated macrophages and their resident CNS counterparts, microglia, are the two most prominent types of myeloid cells in the brain. Together, they often account for more than 40% of the total tumor mass.^30^ As mentioned, they have traditionally been classified as either M1 or M2 types of macrophages, being respectively pro‐ and anti‐inflammatory and characterized by distinct immune markers (such as CD80 and CD86 for M1 or CD163 and CD206 for an M2 subtype). However, the latest evidence suggests that this dichotomy is oversimplified, and we should rather consider these macrophages as a continuum.^59^ The M2‐type microglia were found more frequently in STS,^60^ as one would assume given the immunosuppressive and thus pro‐tumoral function. Since M2‐like microglia/macrophages are more resistant to therapy in GBM, it is speculated that adjuvant therapy even selects for M2 macrophage populations.^61^ Interestingly, it was found that bone‐marrow‐derived macrophages exhibit a strong immunosuppressive function in the tumor center, as opposed to resident microglia.^34^

One study, based on 10 patients from the Chinese Glioma Genome Atlas (CGGA) database, found that reduced accumulation of microglia in the tumor microenvironment was associated with exceptional survival as these tumors were more susceptible to temozolomide (TMZ)‐induced damage. As an underlying mechanism, it was found that in high‐grade astrocytoma, mainly GBM, interleukin 11 (IL‐11) secreted by microglia activates STAT3‐MYC signaling. This leads to the induction of a stem cell state and thereby enhances intra‐tumor heterogeneity and thus resistance to TMZ.^18^, ^62^ Pharmacological inhibition or genetic inactivation (via CRISPR‐Cas9) of this pathway in mice resulted in exceptional survival.^26^, ^63^ However, these findings were not described specifically for LTS, but for above‐median survival in general.

#### 3.2.3 The main cellular players—T‐cells

In GBM T‐cells are much less prevalent than macrophages. GBM is considered a ‘cold’ tumor with low numbers of natural killer cells (NK) and infiltrating T lymphocytes (TILs). These T‐cells, when able to infiltrate the tumor, usually adopt a severely dysfunctional phenotype. Under normal circumstances, our body prevents autoimmunity through tolerance mechanisms. In addition to central tolerance (during the development phase in the thymus), there is a mechanism of peripheral tolerance consisting of peripheral suppression and deletion of cytotoxic immune cells by Treg. GBM hijacks this mechanism preventing an adequate antitumor immune response.^64^ Interestingly, however, there is no correlation between the level of Treg infiltration and survival of GBM patients, suggesting several alternative mechanisms (e.g., MDSCs).^65^

Apart from Tregs, additional evidence points to a pivotal role for T‐cells in LTS, as several genes responsible for specific T‐cell functions were found to be upregulated in LTS compared to STS,^18^ even though this resulted from bulk transcriptomic analyses warranting some caution. Classically, helper T‐cells (Th cells), bearing the potential to further ignite an immune response by interacting with MHCII‐presented peptides on professional antigen‐presenting cells (APCs), are dysfunctional in GBM. This is caused primarily by immunosuppressive cytokines (e.g. Interleukin 10, IL10) and inhibitory co‐receptor interactions (Lymphocyte‐activation gene 3, LAG3; Programmed death‐ligand 1, PDL1).^31^ Th cells differentiate into various subtypes, with Th1 and Th2 best described. A shift to either a Th1 or Th2 type can be influenced by several factors, with the amount of IL‐12 production by the myeloid DC (mDC) being of great importance.^66^, ^67^ In GBM, the balance between Th1 and Th2 has been found to be prognostic, with a Th2‐low balance, associated with the downregulation of the PD‐L1/PD‐1 axis, being correlated with better prognosis.^32^, ^68^ This is similar to what is found in other tumors where a shift toward a Th1 subpopulation favored prognosis.^69^, ^70^ Th2 cells release Il‐4 and IL‐10. These have immunosuppressive functions and thus contribute to tumor progression.^69^ Moreover, differentiation of T‐cells to Th2 cells is seen in GBM, but is hardly been detected in longer surviving GBM patients.^71^ We would also expect Th1 responses to be more in longer living GBM patients, as implied by the presence of several markers like STAT4.^72^, ^73^ As is the case in other malignancies, the activity of cytotoxic T‐cells (CTLs) was found to be an important contributor to long‐term survival.^18^ Despite the high number of exhausted CTLs in the GBM TME, CTLs can be activated by resident microglial cells via the TLR2‐MHC‐I axis.^74^ In a study looking at CD8+ T‐cell infiltration at first presentation, a more pronounced T‐cell infiltration was observed in longer survivors of GBM compared with STS; although results in different studies and approaches used to measure this feature vary.^75^, ^76^ However, T‐cells were quantified histologically in resection specimens from the first surgical intervention, and the T‐cell function (exhausted or activated) was not taken into account. Long‐term survival in the latter study was defined as a survival of more than 403 days, making it hard to estimate the impact of (e)LTS because of our more stringent cut‐off value for LTS. It is also worth mentioning that the mean age in the LTS group (54.3 years) was younger than that in the STS group (65.2 years). Another interesting finding in GBM patients surviving more than 3 years is the fact that the expression of T‐cell and myeloid‐lineage‐associated genes were both elevated.^18^ This was, however, not investigated in true (e)LTS. Multivariate analysis in this study showed that increased immune cell infiltration was correlated with better survival. The authors, however, generalized immune infiltration as one homogeneous entity, referred to only one other study with similar findings (but used a two‐year cut‐off to define long‐term survival), and did not provide the link between the genes discovered and an exact (immunological) function.^77^ With the complexity of the TME in mind, one could argue that this explanation appears an oversimplification.

We would like to highlight a particular case report of an adult female GBM patient, diagnosed with Lynch syndrome, who achieved an overall survival over 81 months.^78^ Lynch syndrome, also known as hereditary nonpolyposis colorectal cancer, is a multi‐tumor syndrome caused by autosomal dominant germline mutations in DNA mismatch repair (MMR) genes. Brain tumors occur in 14% of all patients with Lynch syndrome.^79^ She was treated with anti‐PD‐1 therapy after relapse. Both samples from the primary tumor and recurrence contained high numbers of CD163+ cells, especially in the tumor margins compared to the center of the tumor.^78^ This is in addition to large numbers of CD8+ memory T cells and sustained activation of CD4+ T‐cells. No other markers for macrophages were included in this study (neither a pan‐macrophage marker such as CD68 nor an M1 type marker). Unfortunately, this makes it impossible to distinguish between M1 and M2 macrophages. In the primary sample, CD3+ and CD8+ T‐cells were homogeneously distributed. in the recurrent sample, however, a higher density of CD8+ T‐cells was seen, next to infiltration of persistently activated CD4+ T‐cells. CD8+ T‐cell generation was thus mainly found in the recurrent sample. A similar case report describes a survival of more than 5 years in a patient with a GBM IDHwt who was treated with anti‐PD1 immunotherapy (nivolumab) in combination with radiotherapy.^80^ However, as with all case reports, one should keep in mind that negative results are far less likely to be published. Also, large negative trials with nivolumab have been published in both primary and recurrent settings.^81^, ^82^ In our experience, in some patients with Lynch syndrome, the associated malignant glioma does not respond at all to anti‐PD1 treatment. More research is needed, especially since the initial case series of immunotherapy in (recurrent) MMR‐deficient gliomas suggested no significant effect.^83^, ^84^

Sun et al. investigated the relationship between neoantigens and LTS.^85^ Neoantigens are mutated antigens specifically expressed by tumor tissue while not present in normal cells. They are generated by mutated proteins that form new epitopes. These neoantigens are presented to CD8+ T‐cells, eliciting specific T‐cell responses and a stronger anti‐tumor response. A deep learning model developed by this research group used specific neoantigen compositions to separate LTS from STS using data from The Cancer Genome Atlas (TCGA) database. Importantly, only the combination of increased levels of high‐quality neoantigens combined with increased infiltration of CD8+ T‐cells was associated with long‐term survival in GBM.^76^ Immunotherapies against these neoantigens showed promising preclinical results in lung cancer, colorectal cancer, and melanoma^86^, ^87^, ^88^ Also GBM neoantigen vaccination showed an increased intratumor T‐cell response.^89^ It is important to note that radiotherapy induces immunomodulatory effects through, for example, upregulation and modulation of neoantigen expression.^90^ In general for all tumors, tumor neoantigen load is known to predict clinical response to immune checkpoint inhibitors.^91^, ^92^, ^93^ This could also be a (partial) explanation for the aforementioned case report in which beneficial results were seen for immunotherapy (directed against PD‐1) in combination with radiotherapy, as well as the negative effects seen in larger trials with nivolumab.^80^, ^81^, ^82^

So far, we could state that broadly speaking an increase in CD8+ T‐cells seems to be a returning feature of LTS. However, it also seems clear that further characterization of T‐cells and their state (activated, exhausted) is needed to unravel this complex story.^18^, ^76^

OX40, a T‐cell activation marker, and its ligand OX40L are part of the TNFR/TNF superfamily and are mainly expressed on activated CD4+ and CD8+ T‐cells and antigen‐presenting cells respectively; and the latter also on GBM cells.^94^, ^95^ Costimulatory signaling of OX40 to a T‐cell evokes several key functions for long‐lasting antitumor immunity, mainly promoting Th‐cell generation and activation, cytotoxic T‐cell expansion and survival, and blocking Treg activity.

In both human and mouse GBM samples, OX40 and OX40L were associated with longer survival through CD4 T‐cell activation and thus with antitumor immunity.^95^ Under hypoxic conditions, however, OX40 led to activation of Treg. Another study indirectly confirmed these findings, as IDO deficiency in glioma reduces Treg recruitment and increases survival. This is consistent with the study we discussed earlier.^42^ An increase in IDO is, therefore, associated with Treg recruitment.^96^ This leads to tumor growth and disease progression.

Both Sonoda et al. and Jiang et al. found no differences in Ki‐67 between LTS and STS.^16^, ^17^

To summarize, LTS is associated with an abundance of CD8+ T‐cells that could contribute to a stronger antitumor response. Although T‐cell activation status to our knowledge has not been investigated in true LTS, an increase in activated T‐cells (OX40+) was associated with increased survival in patients. Furthermore, a predominance of M2‐type macrophages was linked to STS, whereas to our knowledge no studies have been published on the M1‐M2 relationship in LTS. More studies are needed to unravel the cell–cell interactions, as all these reports suggest a complex and tumor subtype‐specific cellular correlation between the presence of immune cells in the TME and patient survival.

### 3.3 Molecular features of the tumor microenvironment associated with long‐term survival

#### 3.3.1 The main molecular players—introduction

Several characteristic molecular aberrations have been described in GBM, including chromosome 7 amplification and 10 deletions, mutations of *IDH1&2*, tumor protein p53 (*TP53*), platelet‐derived growth factor receptor alpha (*PDGFRA*), epidermal growth factor receptor (*EGFR*), neurofibromatosis type 1 gene (*NF1*), telomerase reverse transcriptase gene promotor (*TERTp*) and *PTEN*.^97^ As of 2021, WHO CNS tumor classification the term ‘GBM’ is reserved exclusively for grade 4 IDHwt glioma. Moreover, despite the absence of histological features of high‐grade malignancy (necrosis, angiogenesis), an IDHwt glioma can still be classified as GBM when EGFR amplification, the combination of gain of chromosome 7 and loss of chromosome 10 (7+/10‐), and *TERT* promoter mutation are present. This highlights the growing importance of molecular features of brain tumors, particularly GBM.

In GBM, *TP53*, *PDGFRA*, *PTEN*, *TERTp* gene, and *EGFR* are the major driver genes.^98^, ^99^, ^100^ *NF1*, a tumor suppressor gene, and RAS‐GTPase are causally linked to the acquisition of the mesenchymal subtype in GBM, promoting cell invasion, proliferation, and tumorigenesis.^101^, ^102^ Mutations in *TERTp* lead to increased *TERT* expression and de novo telomerase activity in a cancer cell.^103^, ^104^ The *MGMT* gene is a DNA repair enzyme. It rescues tumor cells from alkylating agent‐induced damage, leading to resistance to chemotherapy with alkylating agents.^105^ Epigenetic silencing of the promotor region of this gene results in reduced DNA repair and increased therapy sensitivity.

Overall, it is important to keep in mind that with the current increase in omics data, and thus the discovery of especially passenger mutations, the concern rises that correlation will be mistaken for causality.^106^

#### 3.3.2 Genetics—somatic mutations

A study by Lu et al. tried to identify unique mutations in LTS patients, and thus not present in STS. They analyzed the TCGA database and discovered a set of 10 somatic mutations associated with this subset of LTS.^107^ IDH1 (and IDH2) were the most predictive for long‐term survival, once again supporting the idea of considering IDHmt grade IV glioma as a distinct category in the 2021 WHO classification of central nervous system (CNS) tumors.^14^

Cantero et al. showed that alterations in the PI3K pathway, which is commonly altered in IDHwt GBM, can be found in all LTS patients. However, no uniform abnormalities in this pathway were observed, and PI3K pathway alterations were also seen in STS and are, therefore, rather aspecific.^15^, ^108^

In a small group of seven IDHwt and IDHmt GBM (3 and 4 patients respectively), no prognostic mutations were found within GBM in *ATRX*, a chromatin remodeling protein whose main function is the deposition of histone variant H3.3, and *TERT*. This is in contrast to the significant prognostic effect of *MGMTp* methylation seen in this group.^109^, ^110^ *MGMTp* methylation will be further discussed in the next section on epigenetic alterations.

*TP53* is a gene that plays a key role in the cellular response to DNA‐damaging agents. Mutations lead to the accumulation of p53 protein. Older studies linked *TP53* mutations to improved survival in GBM patients which they attributed to an increased sensitivity to adjuvant chemo‐and radiotherapy,^111^, ^112^ although more recent studies contradict this.^113^

Three distinct cases reported the presence of *TP53* and *PTEN* mutations in LTS, including a case of a woman surviving for 6.5 years with metachronous GBM with a *TP53* and *PTEN* mutation in both tumors.^114^, ^115^, ^116^ In a case report on an eLTS with a giant cell GBM, *TP53* was also mutated.^117^ This latter case dates back to 2001 and the presence of an IDH mutation was not analyzed at this time. The presence of *PTEN* alterations contrasts with previously cited evidence in larger studies, where it was described as an unfavorable prognostic factor.^118^ This latter finding is also confirmed by a study by Sonoda et al.^17^ They found that their STS patients more frequently harbor a phosphatase and tensin homolog (*PTEN*) mutation compared to LTS patients.^17^ This was linked to a distinct TME with less T‐cell infiltration.^119^, ^120^ Although due to a small cohort size, this difference in *PTEN* mutation was not significant. Sonoda et al. also found higher p53 protein expression in LTS, despite no significant differences in *p53* gene mutation rate between LTS and STS were noted.^17^ Hartmann et al. found fewer *TP53* mutations in LTS (10.5%) compared to three‐year survivors (17.5%), but compared to STS (14.5%) the results were not significantly different. In an older study by Deb et al. 4 out of 5 LTS cases tested for p53 expression were positive.^121^

Another commonly mutated gene region in glioma is the promotor of the *TERT* gene. Although *TERT* promotor mutations are associated with worse survival in the general GBM population, no statistically significant differences were seen in LTS GBM.^122^ Of note, it was also found that the *TERT* promotor mutation lost its prognostic value in GBM that were macroscopically completely resected and treated with TMZ.^123^

In one study, three younger adult patients harbored a *H3F3A* gene mutation (encoding the histone variant H3.3). This was either a *K27M* mutation (in two patients), which was linked to shorter overall survival (OS), or in one patient a *G34R* mutation (with an OS of 56 months).^15^ However, since the 2021 WHO CNS tumor classification these tumors categorize as diffuse midline/hemispheric glioma instead of GBM.^14^

#### 3.3.3 Genetics—single nucleotide polymorphism (SNP) and copy number variation (CNV)

The total number of CNVs would be a prognostic factor in high‐grade glioma (but more in IDHmt glioma), suggesting mutations in genes for genomic instability as a possible mechanism behind it.^124^

Several CNV probes predicted LTS, with the deletion of oncogene dual specificity phosphatase 28 (*DUSP28*) having the highest correlation with LTS.^107^ *DUSP28* is part of a family of 25 DUSP genes, responsible for the dephosphorylation of proteins with serine/threonine residues and tyrosine residues, important in cell signaling networks.^125^ A positive correlation between haptoglobin‐related protein (*HPR*) CNVs and LTS was also found. This gene encodes for haptoglobin, a blood plasma glycoprotein, and acute‐phase protein.^126^ Expression of HPR epitopes was previously reported as a predictor of recurrence in breast cancer.^116^ Another interesting discovery in this later study of Lu et al. was that higher expression and amplification of the signal transducing adaptor molecule (*STAM*) gene, encoding for a protein that sorts ubiquitinated membrane proteins for lysosomal degradation, was also associated with LTS. Both in terms of gene expression and CNV analysis.

Another study comparing LTS to STS found no significant differences in CNV burden in either group, except for chromosome 11 (less CNV burden in LTS).^127^ However, CNVs varied in size and location, indicating that there are no universal changes in LTS.

Lu et al. discovered eight predictive single nucleotide polymorphism (SNP) genotypes in their LTS cohort.^107^ Beta‐1,3‐Galactosyltransferase 5 (*B3GALT5*) *gene* (a beta‐galactosyltransferase gene that is shown to be negatively correlated with survival in breast cancer and hepatocellular cancer) and the Trimethylguanosine Synthase 1(*TGS1*) gene were the strongest predictors of extended survival, yet the absolute prognostic value remained weak. This suggests that wild‐type genotypes at these loci are weakly predictive of LTS.^107^, ^128^, ^129^

#### 3.3.4 Epigenetics—methylation profiling

DNA methylation is thought to be one of the strongest predictors of long‐term survival, even stronger than age.^107^ *MGMTp* methylation is a well‐known, favorable prognostic parameter in the general GBM population, as it is a predictive marker of sensitivity to alkylating chemotherapeutic agents like TMZ.^130^ Although it is linked to superior survival in treated GBM patients, one study constituted that after 5 years of survival, no significant difference in predictive value was seen between *MGMTp* methylated and unmethylated GBM patients.^131^ On the other hand, two distinct cases of GBM, one that survived for 7 years and one surviving for more than 20 years, showed *MGMTp* methylation.^115^, ^116^ Another study showed that *MGMTp* methylation was more common in LTS, but no universal patterns were seen associated with survival.^127^ This was also found in eLTS with both IDHmt and IDHwt patients after conducting a multiple logistic regression analysis.^109^ The same was true in a group of 44 LTS (both IDHwt and IDHmt) patients (70.6% vs. 34.9% in STS, *p* < 0.001).^16^

Depending on the statistical model used, between 38 and 43 methylation probes were discovered.^107^ We would like to highlight LETM1 Domain Containing 1 (*LETMD1*), an oncogene, as well as cyclin‐dependent kinase inhibitor 1B (*CDKN1B*), a known tumor suppressor gene. For both genes, hypermethylation was positively correlated to long‐term survival. This is opposed to *TNS4*, where hypermethylation was strongly negatively correlated with LTS. No overlap was seen between the methylation of these prognostic areas seen between LTS and STS. A recent study confirmed that a difference in methylation was linked to survival in GBM.^132^ Although not specifically studied in LTS, hypermethylation of a subset of foci (known as cytosine‐phosphate‐guanine island methylator phenotype (G‐CIMP)) in glioma is in general strongly associated with IDH mutation states, younger patient age, and better survival.^133^, ^134^

#### Transcriptomics

Over the past decade, several classification systems have been proposed in an attempt to define different molecular GBM subgroups. The Verhaak classification, based on transcriptomic data, had an enormous impact on the molecular classification of GBM and is still widely used in the research community today.^135^ Other more recent (RNA‐based) classifications are those of Neftel et al. and Wang et al.^136^, ^137^ No clear one‐on‐one relationship has been found between long‐term survival and any of the four gene expression‐based molecular subtypes of GBM (proneural, neural, mesenchymal and classical).^135^ The heterogeneity of GBM is again evident in a comparative study in which it was seen that peritumoral and deep tumoral regions could be differently classified.^138^ The LTS patient in this study had a proneural composition in the deep tumor area and was classified as neural in the peritumoral area. This composition was more common in, but not unique to, an LTS GBM.

Another study identified four genes associated with exceptional responders. In particular, SH3 domain‐containing GRB2 like 2, endophilin A1 (*SH3GL2*), and ethanolamine‐phosphate phospho‐lyase (*ETNPPL*) were more often expressed in the LTS group. These genes were associated with an increase in ‘synaptic vesicle uncoating’ and ‘intracellular transport’, respectively. Another study on SH3GL2 in glioblastoma confirmed its role in suppressing glioma cell migration and invasion.^139^ Loss of SH3GL2 may therefore contribute to migration and invasion of glioma cells. ETNPPL protein overexpression reduces glioma stem cell growth. Mutations in the encoding *ETNPLL* gene, resulting in reduced ETNPPL protein expression, promote gliomagenesis.^140^

As mentioned previously, genes associated with increased effector immune functions are associated with long‐term survival, underscoring the important role of the host immune system in antitumor response and survival.^18^ This applies to several T‐cell functions such as T‐cell immunological synapse (CD2, CD3D, CD3E, CD3G, CD8B, TCRGC2, TRBC1, TARP, and TRAT1), cytotoxic mediators (Granzyme B, H, K, and M) and markers restricted to activated T‐cells (CD69, ZAP70, CARD11, and VAV1).

EGFR, a transmembrane receptor tyrosine kinase enriched in the classical subtype, and p27, a kinase inhibitory protein linked to inhibition of GBM growth, invasion, and neoangiogenesis, were found to play no role in the immune response in all five LTS in a 2005 study that looked for clinicopathological and expression data linked with more than five‐year survival.^121^, ^141^, ^142^, ^143^

MiR‐222 is the only miRNA found to be predictive for LTS.^107^ The upregulation of this miRNA is associated with a decrease in the p53‐upregulated modulator of apoptosis (PUMA) protein, an inducer of massive apoptosis, and thus regulates mitochondrial pathway and reduces tumor size.^144^ Furthermore, PUMA has been reported to be a proangiogenic factor required for microglial cell survival and proliferation and promotes angiogenesis.^145^ To our knowledge, no association between PUMA and survival in human GBM has yet been reported.

Although several characteristic fusions in GBM, such as *FGFR‐TACC*, *NTRK*, *FIG‐ROS1*, *EGFR‐SEPT14*, and *PTPRZ1‐MET* fusions are linked to oncogenesis regulation and an associated decrease in natural survival, we found no articles reporting a correlation with true (e)LTS.^146^, ^147^, ^148^, ^149^ However, variants of tropomyosin receptor kinase A/B/C (TrkA/B/C), encoded by NTRK fusion mutations seem a promising target in GBM.^150^ Although reported to be extremely rare (<1% of GBM) preclinical studies and clinical trials in this GBM subgroup are promising.^151^ Future trials are needed.

Of note, a study by Lu et al. found a rather weak agreement between methylation and expression profiles in LTS GBM. Furthermore, in Principal Component Analysis (PCA) scatterplots and hierarchical clustering analysis of LTS GBMs, weak agreement of primary GBM was seen with both low‐grade glioma (LGG) and with secondary GBM (arising from a low‐grade lesion). All but one of the 28 GBM were primary GBMs and thus not arising from a former LGG. We could, therefore, argue that LTS GBMs represent a unique subset of GBM rather than a misclassification of LGG or a mixed entity with low‐grade elements. However, this was not true for astrocytoma grade 4 IDH1mt, whose expression profile indeed resembled that of LGG. This study did not identify a unique set of molecular markers to distinguish LTS GBM from other GBM.

### 3.4 Exceptional responders

Several studies identified exceptional therapy responders, but only one described unique patient‐specific molecular data.^78^

In non‐responders to PD‐1 immunotherapy, GBM more often harbors a phosphatase and tensin homolog (*PTEN*) mutation which was linked to a distinct TME with less T‐cell infiltration.^119^, ^120^ As previously mentioned, the same trend was found in LTS‐patients of Sonoda et al. although due to a small cohort size, no significance was relative to STS.^17^ A possible explanation is that in all tumors genomic loss of PTEN is associated with decreased Th1 and CD8+ T‐cells, and increased Th2 cells; and therefore with an immunosuppressive state of the TME.^152^ Moreover, the study by Sonoda et al. established an apparent reciprocal relationship between tumor cell cycle activity and the host's immune functions. This points to the hypothesis that a balance exists between tumor mechanisms on the one hand and host antitumor defenses on the other. Therapeutic interventions could shift this balance in favor of the antitumoral host response.

Another study found a missense mutation in only one gene, *FLG* (encoding for the key protein filaggrin which is a filament‐associated protein that binds to keratin fibers in epithelial cells), in 6 GBM patients with an exceptional response.^10^ The survival of the exceptional responders in this study by Wipfler et al. ranged from 2.4 to 10.6 years. However, this finding seems to us to be a passenger mutation, rather than a driver mutation, since mutations in this gene are among the most common single‐gene alterations, affecting up to 10% of northern Europeans; and its function (as part of the barrier function of the skin) is not directly related to GBM tumorigenesis.^153^, ^154^

The study by Wipfler et al. found 1201 GBM‐associated CNVs, of which only six were present at significantly higher levels in non‐exceptional therapy responders (a very heterogeneous group as the given treatment modality differed between patients). All these genes were located adjacent to genes encoding for EGFR and the tumor suppressor genes cyclin‐dependent kinase inhibitor 2A&B (*CDKN2A* and *CDKN2B*). Only for the latter gene, non‐exceptional responders were more likely to have a loss or deletion.

## 4 CONCLUSION

In this literature review, we summarize the so far understudied cellular and molecular prognosticators associated with (extreme) long‐term survival and exceptional therapy response in GBM patients.

Regarding cellular analysis, several trends are found in €LTS: in general, it can be said that a more profound immunological response with a higher cytotoxic (CD8+) T‐cell infiltration, more activated T‐cells, and less immunological inhibition by Tregs, has inverse correlation with tumoral development and expansion. A higher load of activated cytotoxic T cells was seen €(e)LTS compared with STS. While some T‐cell subsets indicate moderate antitumor capacity, myeloid cells in GBM generally behave protumorally. However, this statement needs to be qualified. For example, lower microglial density was found to be a favorable prognostic factor, as it leads to less stem cell‐induced heterogeneity and thus less therapy‐resistant cellular subsets.^46^

In terms of molecular features, the spectrum of possible underlying alterations is extensive. This should be kept in mind when analyzing reported molecular variations between LTS and STS, as confounding rather than true causal prognosticators are much more likely to occur (this is also true, probably to a lesser extent, for the cellular analysis). It may also explain the variation between studies in the observed molecular features associated with LTS. Regarding *IDH* mutations and *MGMTp* methylation, many studies are available. *IDH* mutations are associated with favorable outcomes and, therefore, IDHmt grade 4 astrocytomas are no longer classified as GBM according to the 5th edition of the WHO CNS classification.^14^ However, IDHwt, which is in general more numerous than its mutated counterpart, is also more frequently encountered in LTS.^155^ *MGMTp* methylation is reported to be a favorable prognosticator in GBM. However, this finding cannot be readily extrapolated to LTS patients, as the evidence in these patients is more controversial.^109^, ^127^ Furthermore, p53 expression, but not *TP53* mutations, was found to be a favorable prognosticator in LTS, possibly due to better treatment response if present. In contrast, PTEN mutations were considered an unfavorable prognostic factor and were more common in STS, although not specific to this subgroup.

As an epigenetic characterization of tumors is clearly gaining momentum to be included in clinical evaluation, future discoveries are likely to lead to more consensus on epigenetic prognosticators in GBM patients. For now, suffice it to say that hypermethylation of genomic regions in GBM is associated with improved survival.

The prognostic value of single markers to predict LTS is clearly of limited value, as evidenced by the diverse findings in the previous sections. The importance of a combined model, integrating multiple levels of analysis, was shown by Lu et al.^107^ They observed, for example, that caveolin 1 (*CAV1*), a tumor suppressor gene, was a much stronger predictor in a pooled regression model.

Exceptional responders tend to express a similar cellular constitution than LTS patients as a higher number of CD8+ T‐cells was seen. However, the number of studies looking at cellular and molecular characteristics in these patients was too limited to draw any conclusions.

We should acknowledge that the great diversity and discrepancy in terms of favorable survival factors in GBM can be at least partially explained by the underlying inter‐and intrapersonal heterogeneity in GBM. This finding explains not only that (molecularly different) GBMs may behave differently in a similar host, or that molecularly nearly identical GBMs may behave differently in a different host, but also that the study of these tumors should attempt to take this heterogeneity into account. This is given that examination of a single sample may not be representative of the whole tumor or cannot always be extrapolated to other GBM. Therefore, the development of an integrated multilevel model based on multiple specimens is preferable to more simplistic models and should be the focus of future research on this prognostic topic.

Another point of interest is the definition of LTS used in literature. It is often defined as a survival of 3 years or even less. This remains a highly arbitrary cutoff with no real clinical correlation, especially from the patient's perspective. Consequently, it seems logical that the molecular and cellular features of these tumors are not significantly different from GBM patients with average survival. Therefore, we advocate defining LTS at a minimum as patients who survive more than 5 years, and eLTS as patients who survive 10 years or more, as these may represent a unique subset of GBM.

Finally, it is important to overcome the many methodological flaws due to overinterpretation of the very scattered data on this topic, often obtained from uncontrolled cohorts. To this end, it seems necessary to investigate the phenomenon of (e)LTS in properly matched and well‐defined patient cohorts to unravel the underlying mechanisms. Currently, there are no individual or combined cellular and/or molecular features that can fully grasp the complexity of extreme long‐term survival in GBM patients.

## AUTHOR CONTRIBUTIONS

**Maxime Vanmechelen:** Conceptualization (equal); formal analysis (equal); methodology (equal); validation (equal); writing – original draft (equal); writing – review and editing (equal). **Paul Clement:** Project administration (equal); resources (equal); supervision (equal); validation (equal); writing – review and editing (equal). **Jean‐françois Daisne:** Validation (equal); writing – review and editing (equal). **Isabelle Vanden Bempt:** Conceptualization (equal); methodology (equal); project administration (equal); supervision (equal); validation (equal); writing – review and editing (equal). **Raf Sciot:** Validation (equal); writing – review and editing (equal). **A. D. Garg:** Validation (equal); writing – review and editing (equal). **Patrizia Agostinis:** Supervision (equal); writing – review and editing (equal). **Frederik De Smet:** Investigation (equal); resources (equal); supervision (equal); validation (equal); writing – review and editing (equal). **Steven De Vleeschouwer:** Conceptualization (equal); investigation (equal); methodology (equal); supervision (equal); validation (equal); writing – original draft (equal); writing – review and editing (equal).

## Data Availability

Not applicable.
